# Virtual Estimator for Piecewise Linear Systems Based on Observability Analysis

**DOI:** 10.3390/s130302735

**Published:** 2013-02-27

**Authors:** Cornelio Morales-Morales, Manuel Adam-Medina, Ilse Cervantes, Luis G. Vela-Valdés and, Carlos Daniel García Beltrán

**Affiliations:** 1 Centro Nacional de Investigación y Desarrollo Tecnológico, Departamento de Ingeniería Electrónica, Interior Internado de Palmira s/n, 69490 Cuernavaca, Morelos, Mexico; E-Mails: adam@cenidet.edu.mx (M.A.-M.); velaluis@cenidet.edu.mx (L.G.V.-V.); cgarcia@cenidet.edu.mx (C.D.G.B.); 2 Departamento de Ingeniería Electrónica y Telecomunicaciones, Universidad Politécnica del Estado de Morelos, Boulevard Cuauhnáhuac #566, Col. Lomas del Texcal, 62550 Jiutepec, Morelos, Mexico; 3 Applied Mathematics Division, Institute for Scientific and Technological Research of San Luis Potosi (IPICyT), Camino a la Presa San José 2055, Col. Lomas 4 sección, CP. 78216, San Luis Potosí S.L.P., Mexico; E-Mail: ilse@ipicyt.edu.mx

**Keywords:** observability, piecewise linear system, estimator, commutation law

## Abstract

This article proposes a virtual sensor for piecewise linear systems based on observability analysis that is in function of a commutation law related with the system's outpu. This virtual sensor is also known as a state estimator. Besides, it presents a detector of active mode when the commutation sequences of each linear subsystem are arbitrary and unknown. For the previous, this article proposes a set of virtual estimators that discern the commutation paths of the system and allow estimating their output. In this work a methodology in order to test the observability for piecewise linear systems with discrete time is proposed. An academic example is presented to show the obtained results.

## 1. Introduction

The automatization of industrial processes currently makes the application of based-model control schemes be more complex, resulting in the need to model this type of systems using piecewise linear systems. In order to solve this problem of complex systems modeling, nowadays new techniques have emerged in order to represent them like piecewise linear systems [1]. The piecewise linear systems are represented by set of linear models, which are commuted through a switching law that allows one to capture the complete dynamics of a system with strong nonlinearities. The commutation between linear models is made through a discreet event or condition that changes the dynamic, so that the system's path evolves in continuous-time fashion [2].

The study and analysis of piecewise linear systems have a strong impact for large-scale systems or systems which naturally exhibit continuous and discrete dynamical behaviors (*i.e.*, hybrid behavior, for example, in electric circuits [3], biological systems [4], electrical machines [5,6] among others). In the literature some works focus on solving the observability problem and state estimation of complex systems through piecewise linear systems like a method in order to simplify the analysis of complex systems [2], this has been a motivation to contribute to addressing piecewise linear systems with a methodology to prove the observability system and the piecewise linear observer proposed in order to help future works to develop more robust and reliable detection schemes and fault diagnosis systems.

Complex systems treated as piecewise linear systems are approached from the design of robust filters for singular [7] systems until Takagi-Sugeno fuzzy systems [8–10], both with delay. Also, in previous works the design of state observers for stochastic systems with uncertainty in the parameters, Extended Kalman filter [11], switched systems with uncertainty in the parameters [12], singular systems with Markovian jumps [13] and autonomous switched systems with state jumps focused by sliding modes [14] are approached. The aforementioned works do not attend to the switching sequence for each dynamic model of piecewise linear system, these works only attend to the time variant delays. In the present work we examine the switching sequence for each dynamic model, which therefore represents a significant contribution to the field of discrete-time piecewise linear systems with unknown active mode varying at arbitrary times.

Discrete-time piecewise linear systems with unknown active mode require tools to solve the problems of observability and state estimation [15–17]. The piecewise linear systems can be classified as: (a) known modes [17] and (b) unknown modes [16]. In the first classification, with system known modes, the estimation and observability problem get simpler because it has *a priori* knowledge of the active mode in all the paths of the evolution system [15,18]. Besides this mode condition allows guaranteeing the asymptotic convergence of error and the observability matrix fulfills with the complete rank condition. However, for the second classification (unknown modes), the estimation problem and observability turn out to be complex due to the lack of knowledge about the active mode on a discrete time *k*.

In order to explain the observability property with known and unknown modes for piecewise linear systems, there are some works that establish the conditions to prove observability, which address the observability problem of discrete-time and continuous-time piecewise linear systems. In [15,17,19], the authors consider known modes switched by periodic switching signals as a function of time; their results are applicable for systems with exogenous inputs, in this same line the authors in [16]address test observability by a finite number of measurements. The previous works do not take into account unknown modes nor when the system is affected by faults or perturbations. In order to face new challenges Babaali y Egerstedt [16] presented a proposal to solve the observability problem for unknown modes (of piecewise linear systems) switched by an arbitrary switching signal. Those authors do not take into account the control input. In the same line this paper proposes a methodology in order to prove observability for piecewise linear systems under conditions of arbitrary switching like a function of system output when it is assumed that sufficient residence time exists for each active mode and the distance between modes is null.

Once the methodology to prove observability in discrete-time piecewise linear systems is established, in this work we propose the design of a piecewise linear observer assuming that initially the system is fully observable under arbitrary commutation, which governs the system dynamics at all time. The advantage of a proposed observer is its capacity to work with unknown system modes and under arbitrary commutation that govern all system dynamics when this is an output function of a discrete-time piecewise linear system. However, other articles only give evidence of state estimation by assuming that the system modes are known and periodically switched [18,20]. In addition, other authors propose the state estimation assuming unknown system modes under arbitrary and periodic switching sequences [16,21–23]. Recent studies as in [22] report a state estimator for a piecewise linear system, where a hybrid observer is proposed considering a commutation sequence of unknown modes like a function of an inputs and outputs system; these results were extended in [21] by calculating the gains of the observer that depend of the mode commutation. Also in [23], the authors propose to estimate the system's states using an observer with unknown input by commutating modes based on the state, and finally in [24] it is restricted to commute only the system's output matrix and estimate the state by using an algorithm of optimization based on an algebraic approach.

The search and the bibliographical review in the piecewise linear system context, particularly in schemes of analysis and observation of observability, sets the guidelines to propose new observation schemes when the modes of the system are unknown, commuted with the commutation law in function of the output and time, in a piecewise linear system on a discrete time. In this context, this paper proposes a methodology to probe the observability and a new approach to estimate the states of a piecewise linear system under the conditions of unknown commutation modes depending on the system's output.

## 2. Approach to the Problem

According the structure of a piecewise linear system in a discrete time:

(1) $$\begin{array}{cc} x_{k+1} & =A(\theta_{k})x_{k}+B(\theta_{k})u_{k}\\ y_{k} & =C(\theta_{k})x_{k} \end{array}$$

where: *x_k_* ∈ ℝ*^n^* is the system's state, *u_k_* ∈ ℝ*^m^* is a known entry of the system, *y_k_* ∈ ℝ*^p^* is the system's output and *θ_k_* is the function of a discrete constant by pieces state that represents the active mode of the system on a discrete time *t_k_* takes its values in the discrete group {1, …, *s*} with *s* being the number of modes that compose the commutes dynamic of the entire system.

In order to define the piecewise linear system's active mode in a discrete time *t_k_* it is expressed *i* ∈ {1, …, *s*} this is possible if *θ_k_* =*i* which corresponds to a specific instant of the system's matrices (*A_i_*, *B_i_*, *C_i_*), with *i* =1, 2, …, *s*. In order to note the commutation time sequence in which every system mode changes it is expressed {*t*_1_, *t*_2, …_*t_k_*} with *k* ≥ 0, those instants of time represent the mode changes that can be established $\theta_{k}\left(t_{i}^{+}\right)\neq \theta_{k}\left(t_{i}^{-}\right)$. For example, taking into consideration *t*_0_ = 0 and the discrete state of the system as *θ*(*t_k_*) = *θ_i_* so *θ_i_* ∈ {1, 2, …, *s*} *t_i_* ≤ *t* ≤ *t_i_*_+1_ with *i* =0, 1, 2, …

To delimit the reach of the investigation, we will work only with autonomous piecewise linear systems, assuming that the time of permanence of each one of the system's modes in active mode is enough to guarantee the mode's observability and that there is no separation time between the transfer of a mode to another, that is to say, it does not represents discontinuousness in the evolution path. The structure of the autonomous piecewise linear system on a discrete time is the following:

(2) $$\begin{array}{cc} x_{k+1} & =A(\theta_{k})x_{k}\\ y_{k} & =C(\theta_{k})x_{k} \end{array}$$

where *x_k_* ∈ ℝ*^n^* is the state's system, *y_k_* ∈ ℝ*^p^* is the system's output and *θk* is the constant discrete state function by pieces that represents the active mode of the system on a discrete time *t_k_* take its values in the discrete group {1, …, *s*} with *s* being the number of modes that share the commuted dynamic of the entire system. From Equation (2) the observability analysis is made in a piecewise linear system in a discrete time and will be treated in further detail in the next section.

## 3. Observability Analysis

In order to define the observability of piecewise linear systems in a discrete time, a finite number of output measurements of the system affected by the commutation law is required. This commutation law allows one to establish commutation sequences for the system's modes, known or unknown, periodical or arbitrary, for the studied case it focuses on commutation arbitrary and unknown sequences. From that, some definitions of observability of linear systems [25] and piecewise linear systems with sequence of known commutation [15] arise. These definitions are:

### Definition 1

A linear system is observable in a time *t*_0_ if its vector of state on that time *t*_0_, can be determined from the output function *y*_“_*_t_*_0_, *_t_*_1”_ (or output consequence), where *t*_0_ ≤ *t*_1_ in some finite time. If this is accomplished for everything *t*_0_, the system is completely observable.

### Definition 2

Piecewise observability. The set of pairs (*A*(1), C(1)), …, (A(*s*), C(*s*)) it is piecewise observable if only exist and integer *N* and all the longitude paths *N* are observables (*i.e.*, *ρ*(*O*)(θ) = *n* where, *ρ*(·) denotes the function of rank and *n* the system dimension.

The previous definitions allow one to extend the analysis of observability to piecewise linear systems with an arbitrary and unknown commutation sequence, which eases establishment of the next proposition:

### Proposition 1

In time invariant linear systems the estimation of states exists if and only if the observability matrix fulfills the complete rank condition, however, for piecewise linear systems this does not apply, because of their different nature, that is to say, it has two inputs: commutation law and control input.

The proposition 1 extended to piecewise linear system guarantees that the observability property is a necessary condition for the system to be observable, the difficulty with this, is that the active mode in a finite time is unknown. In order to resolve this complication it is required that the modes of the system be discerned at all times, so if there are discerned modes the definition 2 can be applied in order to prove the observability in piecewise linear system on a discrete time with a commutation sequence of unknown modes. The use of the definition 2 in the context of piecewise linear systems allows us to establish the next proposition when the system's modes are unknown.

### Proposition 2

In piecewise linear systems invariable in time the estimation of states is done if the matrix of observability fulfills the complete rank condition.

In order to prove the affirmation of the preposition 2 to be true, we need to prove that we count with a sufficient number of measurements of the active mode to guarantee discernibility of mode and observability of state. For that it is assumed that all the system's modes can be known or unknown, depending the type, conditions and system's inputs. In these affirmations, the analysis of observability s presented for the next defined cases based on the amount of measurements of each one of the active modes in a time *t_k_*, this is: (a) *N*_1_ = *N*_2_ = … = *N_s_*; (b) *N*_1_ ≠ *N*_2_ = … ≠ *N_s_*; (c) *N_i_* ≤ *N_j_* ó *N_i_* ≥ *N_j_*, con *i* = 1, 2, … y *j*=1, 2, ….

The three previous cases establish that not all the times of permanence of the active mode will be equal. However, it is assumed that you have a time of permanence sufficient to guarantee the state observability. Besides, it is established that the *s* modes of the longitude system *N* evolve from an instant *k* = 1 until *k*, with *k* ≥ 1.

The dynamic evolution of the subsystem linear group guided by a commutation signal divides the dynamic path of the system in different longitudes, defined by the group of commutation times {*t*_1_, *t*_2_, …, *t_k_*}, with *k* ≥ 0, for example, for the first mode, the time in which this remains active it denoted by *N*_1_, for the second mode beginning in *N*_1_ it is denoted by *N*_2_ and so on until *N_s_*.

Every permanence time of the linear subsystem in active mode allows having a historical view of the evolution of output of the piecewise linear system from the Equation (2) in an instant *k*_=1_ until an instant *k* + *N_s_*. This can be expressed next, beginning for a first active mode with finite time from *k* to *k* + *N*_1_ represented as:

(3) $$\begin{array}{cc} y_{k} & =C(\theta_{k})x_{k}\\ y_{k+1} & =C(\theta_{k+1})A(\theta_{k})x_{k}\\ & \vdots \\ y_{k+N_{1}-1} & =C\left(\theta_{k+N_{1}-1}\right)\prod_{j=2}^{N}A\left(\theta_{k+N_{1}-j}\right)x_{k} \end{array}$$

Equation (3) represents the output measurement of the first active mode of piecewise linear system in a time *k* ≥ 0, where a set of historic data of the dynamic behavior of the linear subsystem is represented by the active mode. However, is there exist *s* linear subsystems with *s* modes of operation then there will be *s* different dwell outputs without a separation time between each mode change, which allows us to create a complete history of the dynamic behavior of the system and it is mathematically expressed by Equation (4):

(4) $$\begin{array}{cc} y_{k}^{1} & =C(\theta_{k})x_{k}\\ y_{k+1}^{1} & =C(\theta_{k+1})A(\theta_{k})x_{k}\\ & \vdots \\ y_{k+N_{1}-1}^{1} & =C\left(\theta_{k+N_{1}-1}^{1}\right)\prod_{j=2}^{N_{1}}A\left(\theta_{k+N_{1}-j}^{1}\right)x_{k}\\ y_{k}^{2} & =C\left(\theta_{k}^{2}\right)\prod_{j=2}^{N_{1}}A\left(\theta_{k+N_{1}-j}^{1}\right)x_{k}\\ y_{k+1}^{2} & =C\left(\theta_{k+1}^{2}\right)A\left(\theta_{k}^{2}\right)\prod_{j=2}^{N_{1}}A\left(\theta_{k+N_{1}-j}^{1}\right)x_{k}\\ & \vdots \\ y_{k+N_{2}-1}^{2} & =C\left(\theta_{k+N_{2}-1}^{2}\right)\prod_{j=2}^{N_{2}}A\left(\theta_{k+N_{2}-j}^{2}\right)\prod_{j=2}^{N_{1}}A\left(\theta_{k+N_{1}-j}^{1}\right)x_{k}\\ y_{k+N_{s}-1}^{2} & =C\left(\theta_{k+N_{s}-1}^{s}\right)\prod_{j=2}^{N_{s}}A\left(\theta_{k+N_{s}-j}^{s}\right)\cdots \prod_{j=2}^{N_{2}}A\left(\theta_{k+N_{2}-j}^{2}\right)\prod_{j=2}^{N_{1}}A\left(\theta_{k+N_{1}-j}^{1}\right)x_{k} \end{array}$$

Equation (4) represents the evolution of piecewise linear system evolution in a time *k* ≥ 0 considering the *s* modes of the concatenate system operation. To compact Equation (4) the next equation can be established:

(5) $$\begin{array}{ll} Y_{N_{1}}^{1} & ≜Q\left(\theta_{N_{1}}^{1}\right)x_{k}\\ Y_{N_{2}}^{2} & ≜Q\left(\theta_{N_{2}}^{2}\right)H\left(\theta_{N_{1}}^{1}\right)x_{k}\\ & \vdots \\ Y_{N_{s}}^{s} & ≜Q\left(\theta_{N_{s}}^{s}\right)H\left(\theta_{N_{s}}^{s}\right)\cdots H\left(\theta_{N_{1}}^{1}\right)x_{k} \end{array}$$

where:

$$Y_{N_{s}}^{s}≜\left(\begin{array}{c} y_{k}^{s}\\ y_{k+1}^{s}\\ \vdots \\ y_{k+N_{s}-1}^{s} \end{array}\right);H\left(\theta_{N_{s}}^{s}\right)≜\prod_{j=2}^{N_{s}}A\left(\theta_{k+N_{s}-j}^{s}\right)x_{k};x_{k};Q\left(\theta_{N_{s}}^{s}\right)≜\left(\begin{array}{c} C\left(\theta_{k}^{s}\right)\\ C\left(\theta_{k+1}^{s}\right)A\left(\theta_{k}^{s}\right)\\ \vdots \\ C\left(\theta_{k+N_{s}-1}^{s}\right)H\left(\theta_{N_{s}}^{s}\right) \end{array}\right)$$

From Equation (5) the observability matrix for a piecewise linear system in a discrete time orchestrated by a commutation law that generates an arbitrary and unknown commutation is defined, this being under the condition $\theta_{k}\left(t_{i}^{+}\right)\neq \theta_{k}\left(t_{i}^{-}\right)$. Then the new observability proposed matrix has the following structure:

(6) $$O\left(\theta_{N_{s}}^{s}\right)≜\left(\begin{array}{c} Q\left(\theta_{N_{1}}^{1}\right)\\ Q\left(\theta_{N_{2}}^{2}\right)H\left(\theta_{N_{1}}^{1}\right)\\ \vdots \\ Q\left(\theta_{N_{s}}^{s}\right)H\left(\theta_{N_{s}}^{s}\right)\cdots H\left(\theta_{N_{1}}^{1}\right) \end{array}\right)$$

Finally, the output vector is defined in the following way:

(7) $${\begin{array}{cc} Y\left(\theta_{N_{s}}^{s},x_{k}\right)≜O\left(\theta_{N_{s}}^{s}\right); & Y_{N_{s}}=\left[\begin{array}{cccc} Y_{N_{1}}^{1} & Y_{N_{2}}^{2} & \cdots & Y_{N_{s}}^{s} \end{array}\right] \end{array}}^{T}$$

The results of the previous analysis give as product the construction of the observability matrix and the understanding of how the commutation law affects the system's dynamic behavior. From that analytic procedure, the next lemma is proposed:

### Lemma 1

If the observability matrix of piecewise linear system defined in Equation (6) fulfills the complete rank condition, it is to say, $\rho \left(O\left(\theta_{N_{s}}^{s}\right)\right)=n$, then it is guaranteed that the piecewise linear system is completely observable, therefore the initial state of the system from the Equation (7) can be rebuilt.

To prove the lemma 1, it is required that assumption 1 be fulfilled, which is:

### Assumption 1

If enough measurements are at hand in order to guarantee the observability and discernibility of the mode, so the piecewise linear system is fulfilled so it is observable and mode discernible.

By accomplishing the assumption 1 the observability and discernibility of mode can be guaranteed, so the sequence of mode gets determined in any instant of time *k* and allows us to rebuild the states, in order to do that, [16] is retaken.

### Proposition 3

If a system is piecewise observable with index *N_s_* and each path of longitude *N_s_* is discernible then the state is completely observable [16]. The demonstration of this proposition it is reported in [16]. Combining the previous cases related to the time of permanence in active mode for each linear subsystem and the analysis of observability sum up in the lemma 1, the preposition 2 gives this diagram.

**Figure 1.**
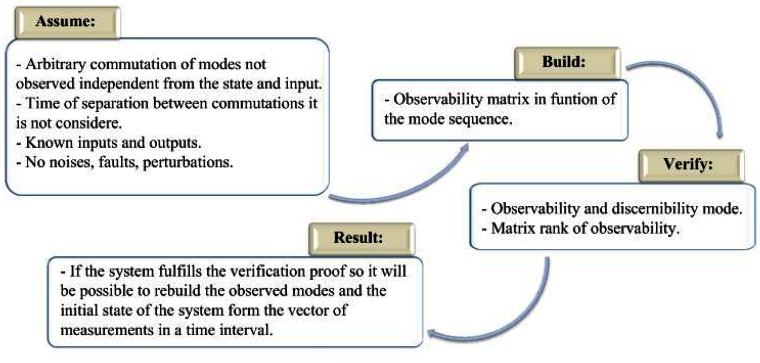
Methodology to prove the observability in piecewise linear systems.

Figure 1 presents a methodology to prove the observability in piecewise linear systems. In the first step the conditions are established, in the second step the observability matrix is built, in the third step the gets verified and in the last step the result is presented. The methodology presented will allow one to prove the observability in piecewise linear systems in a discrete time.

## 4. Design of the Estimator for a Piecewise Linear System Analysis

The design of estimators in piecewise linear systems in a discrete time represents a complex challenge, due in the first place to the fact you must guarantee that the system be completely observable, it is to say, that lemma 1 seen in the previous section is fulfilled. In this context a piecewise linear system taking the structure of Equation (1), with the next conditions: a sufficient permanency time in active mode, the commutation of the modes is arbitrary and is unknown, the time of separation between modes is null, the inputs and outputs are known. From those conditions the design of the observer mentioned before sets the following hypothesis:

### Hypothesis 1

If the piecewise linear system is discernible of mode, and observable in states, so an arbitrary and unknown observer can be designed, so it can estimate the state system. To prove hypothesis 1, it is assumed that the piecewise linear system is discernible in mode and observable in state from a finite amount of measurements in the active mode. This is *Y*(*θ*, *x*) ∈ ℝ (*O*(*θ*)), where ℝ (*O*(*θ*)) denotes the space of column rank of the observability matrix. To ensure the count of the sufficient number of measurements, the system proves that the system is completely observable, but for this, an estimator has to be designed from the active mode in the system to know the time *t_k_* in which a subsystem can be found evolving a linear subsystem. The estimator of the active mode is made based on a comparison of the outputs of each one of the modes, then the estimator structure is:

(8) $$\begin{array}{ll} {\hat{x}}_{k+1} & =A_{i}{\hat{x}}_{k}+B_{i}u_{k}\\ {\hat{y}}_{k} & =C_{i}{\hat{x}}_{k} \end{array}$$

where (*A_i_, B_i_, C_i_*), with *i* =1, 2, …*s* define a family of linear subsystems in a discrete time *t_k_*, which is possible if *θ_k_* = *i*, which corresponds to a specific instant of the system's matrix, *x̂_k_* ∈ ℝ*^n^* is the estimated state of a linear subsystem, *u_k_* ∈ ℝ*^m^* is an entry known to the system, *ŷ_k_* ∈ ℝ*^p^* is the output of a linear subsystem and *θ_k_* is the function of the constant piecewise discrete state.

In order to detect the system's active mode a comparison between the real systems' output minus the output of every one of the linear subsystem is realized on Equation (8), this is defined as:

(9) $${\hat{\theta }}_{k}=y_{k+N_{s}-1}^{s}-C_{i}{\hat{x}}_{k}$$

where *θ̂_k_* is the estimation of the state function of the piecewise constant discrete state, $y_{k+N_{s}-1}^{s}$ is the output of the complete piecewise linear system evolving at all instants of time from Equation (4). From the proposal of the detector of active mode the estimation of states using a linear observer by pieces is made. For that, the following hypothesis is proposed:

### Hypothesis 2

If the outputs are available on each step, it is possible to design an observer on piecewise linear systems in order to estimate a *x̂_k_* from *x_k_* so that, lim*_k_*_→ ∞_ ‖*x_k_*− *x̂_k_* =0‖.

To prove the hypothesis 2 proves that the operation mode represented by the linear mode is observable, for that the use of the methodology proposed in the Figure 1 is required and for the estimation of state of that mode, the error converges asymptotically to zero, so that, if it is considered that a set of modes represented by several linear models conserves the observability and yet manages to design a piecewise linear observer, commuted by a signal of arbitrary and known commutation, to estimate the states.

Based on the analysis of observability presented in the previous section and the detector of active mode of the Equation (9), a piecewise linear observer is proposed inspired on the classic observer of Luenberger, which structure is as follows:

(10) $$\begin{array}{ll} {\hat{x}}_{k+1} & =A\left({\hat{\theta }}_{k}\right)u_{k}+K\left({\hat{\theta }}_{k}\right)\left(y_{k}-C\right)\\ {\hat{y}}_{k} & =C\left({\hat{\theta }}_{k}\right){\hat{x}}_{k} \end{array}$$

where: *K* (*θ̂_k_*) is the matrix of increases from the observer of appropriate dimension and *x̂_k_* is the estimated state in the time *k*, *ŷ_k_* is the estimated output in time *k*, *θ ^_k_* is the estimation of the constant discrete function by pieces.

**Figure 2.**
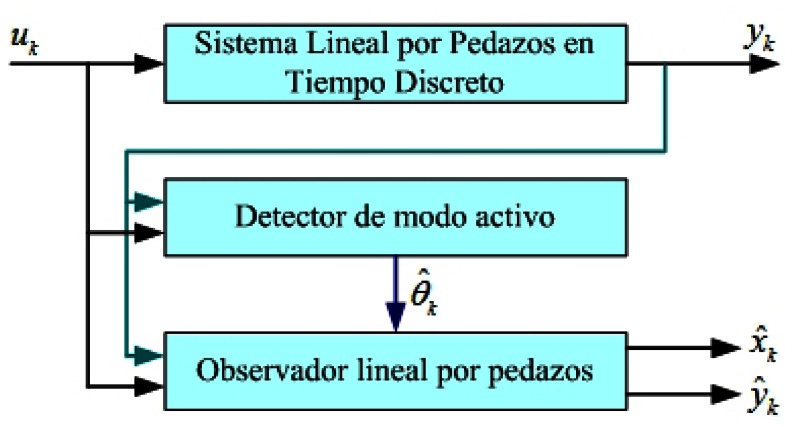
Virtual estimator for piecewise linear systems.

The objective of the piecewise linear observer that is to be build is to reconstruct the estimate state *x̂_k_* from *x_k_* and the output *ŷ_k_* from *y_k_* based in the knowledge of the outputs *y*_1_, …,*y_k_*, and inputs *u*_1_, …, *u_k_*, so that, lim*_k_*_→ ∞_ ‖*x_k_*− *x̂_k_* =0‖. The proposed scheme from the virtual estimator for piecewise linear systems is illustrated in Figure 2.

In the Figure 2 each one of the parts that form an estimator of states and the interaction among them is illustrated, such as they integrate the piecewise linear observer system with sequence of commutation of operation modes of *s* linear subsystems with no variables in time. The previous can be summarized in the next definition:

### Definition 3

The piecewise linear observer converges if lim*_k_*_→ ∞_ ‖*x_k_*− *x̂_k_* =0‖ for all the sequence of inputs, all sequence of modes, all the initial states and estimated initial states.

Based on Definition 3 we assume that we have *N* amount of measurements sufficient and necessary to prove the system's observability, if the condition of complete rank is satisfied, so the error of estimation of state tends asymptotically to zero when *k* → ∞, *i.e.*, lim*_k_*_→ ∞_ ‖*x_k_*− *x̂_k_* ‖ = 0.

From that structure of a piecewise linear observer of Equation (10) and the estimator of mode in Equation (9), we present a academic example in order to validate the results. Thus, *y_k_* y *u_k_* are assumed known with a mode sequence ${\left\{\theta_{k}\right\}}_{k=1}^{\infty }$ arbitrary and unknown.

## 5. Academic Example

In order to prove the theoretical results obtained a numerical example must be presented. For that, let us consider the next piecewise linear system as described in Equation (1), where the set of matrixes of the piecewise linear system *A_i_*, *B_i_* y *C_i_* is:

(11) $$\begin{array}{lll} A_{1}=\left(\begin{array}{cc} 0.6 & 0.10\\ -0.195 & 0.79 \end{array}\right); & C_{1}=\left(\begin{array}{cc} 1 & 1 \end{array}\right); & B_{1}=\left(\begin{array}{c} 0.20\\ 0.20 \end{array}\right);\\ A_{2}=\left(\begin{array}{cc} 0.45 & 0.16\\ -0.36 & 0.545 \end{array}\right); & C_{2}=\left(\begin{array}{cc} 1 & 1 \end{array}\right); & B_{2}=\left(\begin{array}{c} 0.5\\ 0.5 \end{array}\right);\\ A_{3}=\left(\begin{array}{cc} 0.59 & 0.10\\ -0.195 & 0.79 \end{array}\right); & C_{3}=\left(\begin{array}{cc} 1 & 1 \end{array}\right); & B_{3}=\left(\begin{array}{c} 0.6\\ 0.7 \end{array}\right); \end{array}$$

The set of linear models of the Equation (11) represent a family of linear subsystems that integrate a piecewise linear system of second order. This is implemented in simulation with arbitrary unknown reign by a law of commutation in function of output and time commutation sequences expressed as:

Given an initial time *t*_0_, a signal of commutation *s* defined in terms of input and putout in a time interval [*t*_0_, *t*_1_) with *t*_0_ < *t*_1_ < ∞, *_θ_*: [*t*_0_, *t*_1_) ↦ *s* expresses the output in all time, where *s* is the number of modes that represent each one of the linear subsystems.

The validation in simulation of the results with parameters of sequence design of mode unknown reign by the commutation law in function of the output and the time is done first with the test of observability of the linear system by pieces based on Equation (10).

To build the observability matrix of piecewise linear system of Equation (11) it uses the Equation (4) examining that matrix of observability, it was found that rank is complete, that is to say:

(12) $$\rho \left(O\left(\theta_{N_{s}}^{s}\right)\right)=2$$

Once Equation (12) is satisfied, that is to say, that rank of the matrix of observability equals *n* then the piecewise linear observer on a discrete time to rebuild states of the system can be implemented with success, and this observer is also known as a virtual sensor. The design of the virtual sensor in order to rebuild the dynamics of the complete output of the states was made by concatenating each one of the paths of the linear subsystems assuming that the commutation of the subsystems was arbitrary and unknown.

The observer gains are calculated by the pole placement technique, in order to improve the performance and response of the system in comparison with other sophisticated techniques. In order to show the effectiveness of methodology proposed, this is compared with the results in [18]. This methodology considers unknown and arbitrary switching and in [18], the authors consider known and arbitrary switching. In this context, we carried out a simulation of the academic example.

**Figure 3.**
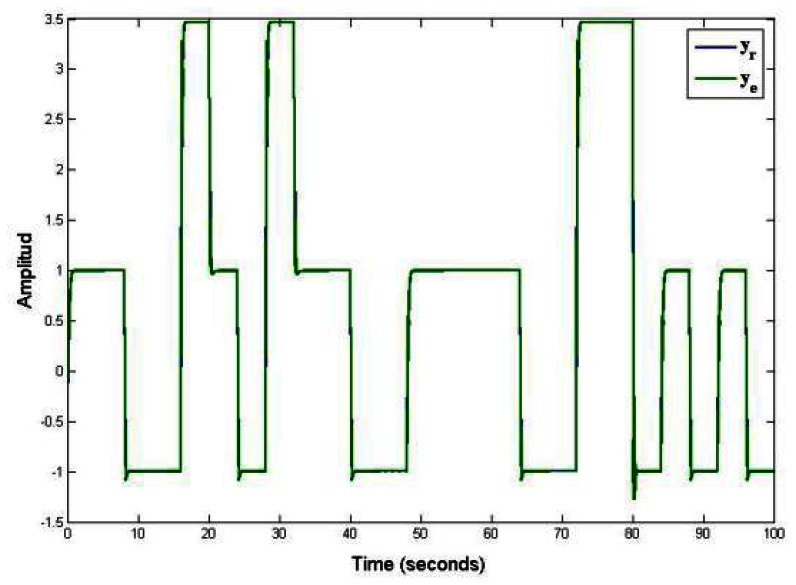
Output of the observer in piecewise a linear system, unknown and arbitrary commutation sequence.

The Figure 3 shows the results of piecewise linear systems governed by an unknown and arbitrary commutation sequence. Figure 3 shown the virtual sensor output (*y_e_*) under an unknown and arbitrary commutation sequence, which is compared with the output of the piecewise linear systems (*y_r_*). The output monitoring system *y_e_* of the piecewise linear systems proposed in Equation (11) is made by using a virtual estimator, whose structure is given by Equation (10). In this example, notice that the output of estimator reproduces with precision the real output of system in spite of the estimation for active mode of system *_θ̂k_*. Where *_θ̂k_* is used to commute the piecewise linear observer.

**Figure 4.**
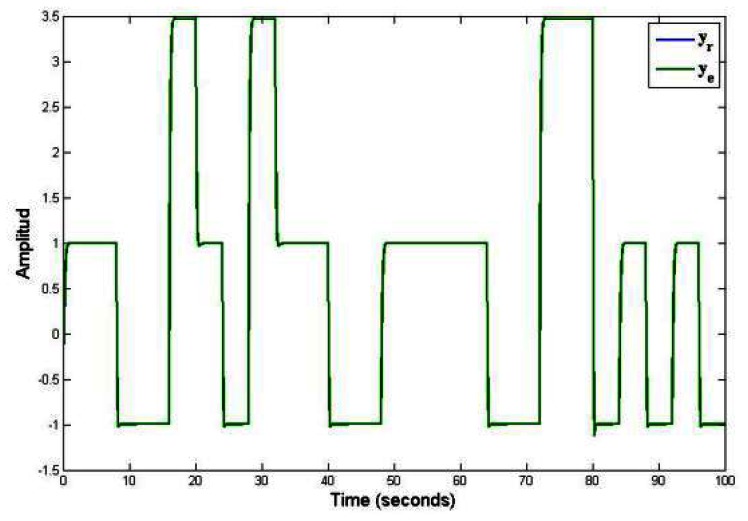
Output of the observer in piecewise a linear system, known and arbitrary commutation sequence.

The Figure 4 shows the behavior of piecewise linear system under a known and arbitrary commutation sequence. In this figure is presented the virtual sensor output (*y_e_*) that is given in [18], where the authors proposed a known and arbitrary commutation sequence in order to commute each of models for an piecewise linear system. In Figure 4 the output of piecewise linear system (*y_r_*) and estimate output by virtual sensor (*y_e_*) are accurate because the commutation sequence is known. The simulation graphs of the system were made with a time of 100 seconds in order to appreciate the dynamic behavior of the piecewise linear system (formed by a family of three linear subsystems). These types of dynamic behaviors appear a lot when different dynamics are combined of several linear systems, for example: Hybrid systems of energy generation, converters, chemical reactors, flight systems, among others.

Figures 3 and 4 shows the response of the discrete—time piecewise linear systems under arbitrary commutation sequence (known and unknown). These figures shown the comparison between our proposed work and the work reported in [18].

**Figure 5.**
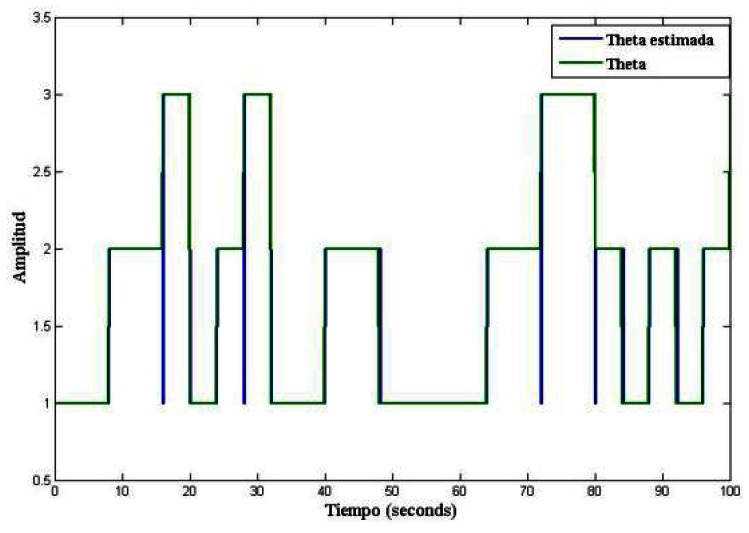
Commutation signal: estimated *vs.* real.

To concatenate each one of the paths of the linear subsystems and implement a piecewise linear observer, it was necessary to estimate the active mode, by using the Equation (9). The evolution of the commutation mode of each one of the linear systems is illustrated in Figure 5.

The proposed technique can be upgraded, for example, to propose schemes of estimation of the more sophisticated modes and assume that the set of all estimated states are enclosed by the commutation law. This will imply a better refinement to the estimation technique in the evolution of states before the change of operation mode, and also a better convergence of the piecewise linear observer.

To quantitatively measure the precision of the proposed approach the mean squared error (MSE) is used here. MSE of an estimator is one of many ways to quantify the difference between values implied by an estimator and the true values of the quantity being estimated. This MSE measures the average of the squares of the “errors”. The error is the amount by which the value implied by the estimator differs from the quantity to be estimated. The results are exposed in Table 1.

The output error of different approaches is approximately to zero. This means that the proposed approach provides satisfactory results, although the observer works with unknown and arbitrary switching sequences.

## 6. Conclusions

This article presented the analysis to prove the observability in a piecewise linear system in a discrete time represented systems ruled by an arbitrary and unknown commutation sequence. Besides, a proposal of a virtual estimator known as piecewise linear observer was presented, that solved the problem with commutation by integrating it an active mode detector. These types of systems are very helpful in the design of new control schemes, or detection of faults based on observers, among others. The obtained results in the estimation of states and the output of the system still can be upgraded by implementing a new detector more sophisticated of mode or by proposing another more elaborate estimator structure. The results encountered in this work can be extended to the design of diagnosis and fault-detection schemes based on observers, where this new design can be implemented in a fault-tolerant control for a piecewise linear system.

## Figures and Tables

**Table 1.** Output error of piecewise linear system.

**Commutation Sequence of Modes**	**Mean Squared Error**
Active mode of known system	0.0069
Active mode of unknown system	0.0966
